# Treg/Th17 imbalance and its association with frailty in elderly patients with community-acquired pneumonia

**DOI:** 10.3389/fmed.2026.1778880

**Published:** 2026-05-20

**Authors:** Chulun Zeng, Yiming Yuan, Qiuyu Han

**Affiliations:** 1 People’s Hospital of Chongqing Liangjiang New Area, Chongqing, China; 2 West China Hospital of Sichuan University, Chengdu, China; 3 The Third Affiliated Hospital of Chongqing Medical University, Chongqing, China

**Keywords:** community-acquired pneumonia, cytokine, frailty, Th17 cells, Treg cells

## Abstract

**Objective:**

To investigate the potential role of Treg/Th17 imbalance in frail elderly patients with community-acquired pneumonia (CAP) by analyzing the clinical characteristics of elderly CAP patients, along with the proportions of regulatory T (Treg) cells and helper T (Th17) cells 17, and related cytokine levels.

**Methods:**

A total of 61 hospitalized CAP patients aged ≥ 65 years were continuously enrolled from June 2021 to January 2022. Frailty status was assessed using the FRAIL scale, and patients were divided into a frail group (*n* = 33) and a non-frail group (*n* = 28). General demographic data, laboratory test results, Charlson Comorbidity Index (CCI), Pneumonia Severity Index (PSI), and hospitalization outcomes were collected. The proportions of peripheral blood Treg and Th17 cells were measured, along with the serum levels of TGF-β, IL-10, and IL-17A.

**Results:**

The frail group exhibited older age, more comorbidities, more severe illness, poorer prognosis, longer hospital stays, and higher treatment costs compared to the non-frail group. Additionally, the frail group had significantly higher proportions of Treg cells and Treg/Th17 ratios, as well as higher TGF-β levels and lower IL-17A levels (*P* < 0.05). Correlation analysis showed a positive relationship between TGF-β levels and Treg proportions, and between IL-17A levels and Th17 proportions. After adjusting for confounding factors, frailty remained an independent factor influencing the elevated Treg/Th17 ratio. Logistic regression analysis indicated that advanced age, high CCI score, high Treg/Th17 ratio, low serum albumin, and female gender were independent risk factors for frailty in elderly CAP patients.

**Conclusion:**

Frail elderly CAP patients exhibit immune imbalance characterized by elevated Treg proportions and Treg/Th17 ratios, which may be associated with their poor prognosis and suggests potential value for immune-targeted interventions. The dysregulation of cytokines TGF-β and IL-17A aligns with the changes in Treg/Th17 proportions, potentially playing a role in the regulatory mechanisms of frailty-related immune imbalance.

## 1 Introduction

Community-acquired pneumonia (CAP) refers to infectious inflammation of the lung parenchyma contracted outside of a hospital, including cases where a patient, infected with a pathogen having a defined incubation period outside the hospital, develops pneumonia during the incubation period after admission. CAP is a major cause of illness and death in the elderly. Globally, it is associated with approximately 2.2 million deaths annually (1). The elderly are particularly susceptible to CAP, largely due to age-related decline in organ function and the cumulative effect of long-term environmental risk exposures. The incidence is especially high among those aged 85 years and above, reaching nearly 10 times the rate observed in younger age groups (2). Supporting this trend, a study of 323,992 pneumonia patients in Hong Kong found that individuals aged 65 and above accounted for 75% of hospitalizations and 90% of fatal cases, representing the majority of related healthcare expenditures (3). In this vulnerable population, frailty—a state of reduced physiological reserve and increased vulnerability—is a critical factor that exacerbates clinical outcomes (4). The prevalence of frailty increases with age, with cross-national surveys reporting rates between 22 and 26% in older populations (5). Its pathophysiological mechanisms may involve factors such as chronic inflammatory responses, immunosenescence, comorbidities, malnutrition, and sarcopenia (6, 7). In the context of acute infections such as CAP, frailty is strongly linked to elevated short-term mortality, prolonged hospitalization, and higher rates of hospital readmission (8–10). Notably, frailty not only correlates with CAP severity but may also increase susceptibility to pneumonia (11). Therefore, understanding the interplay between frailty and CAP is clinically essential.

The pathophysiological link between frailty and adverse CAP outcomes may involve immunosenescence, the age-related dysregulation of immune function. This decline is considered a common mechanism contributing to both the development of frailty and impaired control of infections. A crucial aspect of immune homeostasis affected by aging is the balance between regulatory T cells (Tregs) and T helper 17 cells (Th17). Tregs function to suppress excessive inflammation by secreting inhibitory cytokines such as IL-10, IL-35, and TGF-β, which suppress the proliferation of other immune cells and the production of cytokines (12, 13). Th17 cells primarily secrete IL-17A, playing a significant role in initiating inflammatory responses in the body. The Treg/Th17 balance is vital for an effective and controlled immune response, and its disruption has been implicated in various age-related and infectious diseases.

While the clinical association is well-established, the specific immune profile contributing to poor outcomes in frail elderly patients with CAP remains unclear. In particular, it is unclear whether and how the Treg/Th17 balance, a key axis in immunosenescence, is altered in frail elderly patients with CAP. Existing research has often studied frailty, pneumonia immunology, or immunosenescence in isolation, or has focused on general elderly populations without specifically examining the frail subgroup. This absence of an integrated research approach leaves an important question unanswered. Specifically, exploring whether frail CAP patients exhibit a unique Treg/Th17 imbalance may help explain why they are more vulnerable and tend to have worse clinical outcomes.

Therefore, this study aimed to investigate the potential role of Treg/Th17 imbalance in frail elderly patients with CAP. We conducted a cross-sectional analysis comparing clinical characteristics, peripheral blood Treg and Th17 cell proportions, and related cytokine levels (TGF-β, IL-10, IL-17A) between frail and non-frail elderly patients with CAP. We hypothesized that frail patients would exhibit a specific immune imbalance characterized by Treg/Th17 imbalance, which may be associated with their adverse clinical outcomes.

## 2 Materials and methods

### 2.1 Study population

We consecutively enrolled 61 elderly patients with community-acquired pneumonia (CAP) from West China Hospital, Sichuan University, between June 2021 and January 2022 as the study subjects. Frailty was assessed using the Frailty Screening Scale (FRAIL), and participants were grouped accordingly, with 33 patients in the frail group and 28 patients in the non-frail group.

Inclusion criteria: (1) Age ≥ 65 years; (2) Meeting the diagnostic criteria for CAP; (3) Provision of informed consent and voluntary participation in the study.

Exclusion criteria: (1) Patients with autoimmune diseases, solid tumors, or hematological malignancies; (2) Patients who had used immunosuppressive agents or systemic corticosteroids within 14 days; (3) Patients with infections caused by specific pathogens such as human immunodeficiency virus (HIV), hepatitis B virus (HBV), or Mycobacterium tuberculosis; (4) Patients with incomplete medical records.

This study was reviewed and approved by the Ethics Committee of West China Hospital, Sichuan University. All participants voluntarily took part in the study, were fully informed of the study-related information and precautions, and personally signed the informed consent form.

### 2.2 Study data

(1) General information: age, gender, laboratory test results; (2) Treg and Th17 cell count ratio, as well as levels of TGF-β, IL-10, and IL-17A; (3) CCI and PSI scores; (4) Details such as the length of hospital stay, medical expenses, and clinical outcomes of the patients.

Immunophenotyping of T-cell subsets by flow cytometry. Peripheral blood Treg and Th17 cells proportions were determined using well-established surface marker panels. Treg cells were identified as CD4^+^CD25^+^CD127^–/low^. This phenotype is widely used in clinical immunology research because the low expression of CD127 (IL-7 receptor α-chain) is a robust surface marker that inversely correlates with the suppressive function of human Treg cells (14). Th17 cells were identified by the phenotype CD4^+^CD196^+^CD183^–^. This chemokine receptor-based signature effectively enriches for CD4^+^ T cells that are committed to the Th17 lineage and have the capacity to produce IL-17A, without the need for *ex vivo* stimulation that alters cellular activation states. These surface-staining strategies allow for the specific enumeration of these functional subsets while preserving the native state of the cells, making them particularly suitable for stable and reproducible assessment in clinical cohort studies.

### 2.3 Statistical analysis

SPSS statistical software was used for data analysis. Measurement data conforming to normal distribution and homogeneity of variance were expressed as (mean ± standard deviation), while data not meeting these criteria were expressed as median (P25, P75). For comparisons between two groups, independent samples *t*-tests were used for data with normal distribution; otherwise, rank-sum tests were employed. Correlation analysis for normally distributed data utilized Pearson’s test, while non-normally distributed data were analyzed using Spearman’s test. A *P*-value < 0.05 was considered statistically significant.

## 3 Results

### 3.1 Basic information and laboratory tests

The prevalence of frailty among all study subjects was 54.10%. The average age of the frail group was higher than that of the non-frail group (*P* < 0.05), while there was no statistically significant difference in gender composition between the two groups. Serum creatinine and N-terminal pro-brain natriuretic peptide (NT-proBNP) levels were higher in the frail group compared to the non-frail group (*P* < 0.05), and serum albumin levels were lower in the frail group (*P* < 0.05). No statistically significant differences were observed in other results (Table 1).

**TABLE 1:** Basic information and laboratory tests.

Characteristics	Frail group	Non-frail group	*P*
Age (years)	83.94 ± 7.29	75.93 ± 5.87	<0.05
Gender (male)	21 (63.64%)	18 (64.29%)	>0.05
WBC (*10^9/L)	9.52 ± 4.87	9.72 ± 4.29	>0.05
Creatinine (μmoL/L)	88.00 (69.00, 126.50)	72.50 (60.25, 102.25)	<0.05
Albumin (g/L)	32.74 ± 4.27	35.74 ± 4.69	<0.05
PCT (ng/mL)	0.83 (0.68, 1.17)	0.67 (0.51, 0.87)	>0.05
NT-proBNP (ng/L)	1678.00 (708.50, 4414.50)	1233.50 (249.50, 1964.00)	<0.05

### 3.2 PSI and CCI scores

Both PSI and CCI scores in the frail group were higher than those in the non-frail group (*P* < 0.05). According to PSI risk stratification, the proportion of medium-high risk patients in the frail group was also greater than that in the non-frail group (*P* < 0.05) (Table 2).

**TABLE 2:** PSI and CCI scores.

Characteristics	Frail group	Non-frail group	*P*
PSI score	126.39 ± 25.52	102.18 ± 20.23	<0.05
Risk stratification			
- Low risk	5	11	
- Medium-high risk	28	17	<0.05
CCI score	3.00 (2.00, 4.00)	1.00 (1.00, 3.00)	<0.05

### 3.3 Hospitalization details

Compared to the non-frail group, the frail group exhibited a higher incidence of complications during hospitalization, longer hospital stays, and higher treatment costs (*P* < 0.05) (Table 3).

**TABLE 3:** Hospitalization details.

Complications	Frail group	Non-frail group	*P*
Overall complications	13	4	<0.05
- Shock	4	2	
- Gastrointestinal bleeding	4	1	
- Secondary infection	5	1	
Death	5	0	
Hospital stays	16.00 (11.00, 20.00)	11.50 (9.00, 16.25)	<0.05
Treatment costs	24224.00 (15729.50, 32763.50)	13592.50 (11267.25, 23539.25)	<0.05

### 3.4 Comparison of peripheral blood CD4^+^T, Th17, and Treg cell proportions and Th17/Treg ratio

**FIGURE 1:**
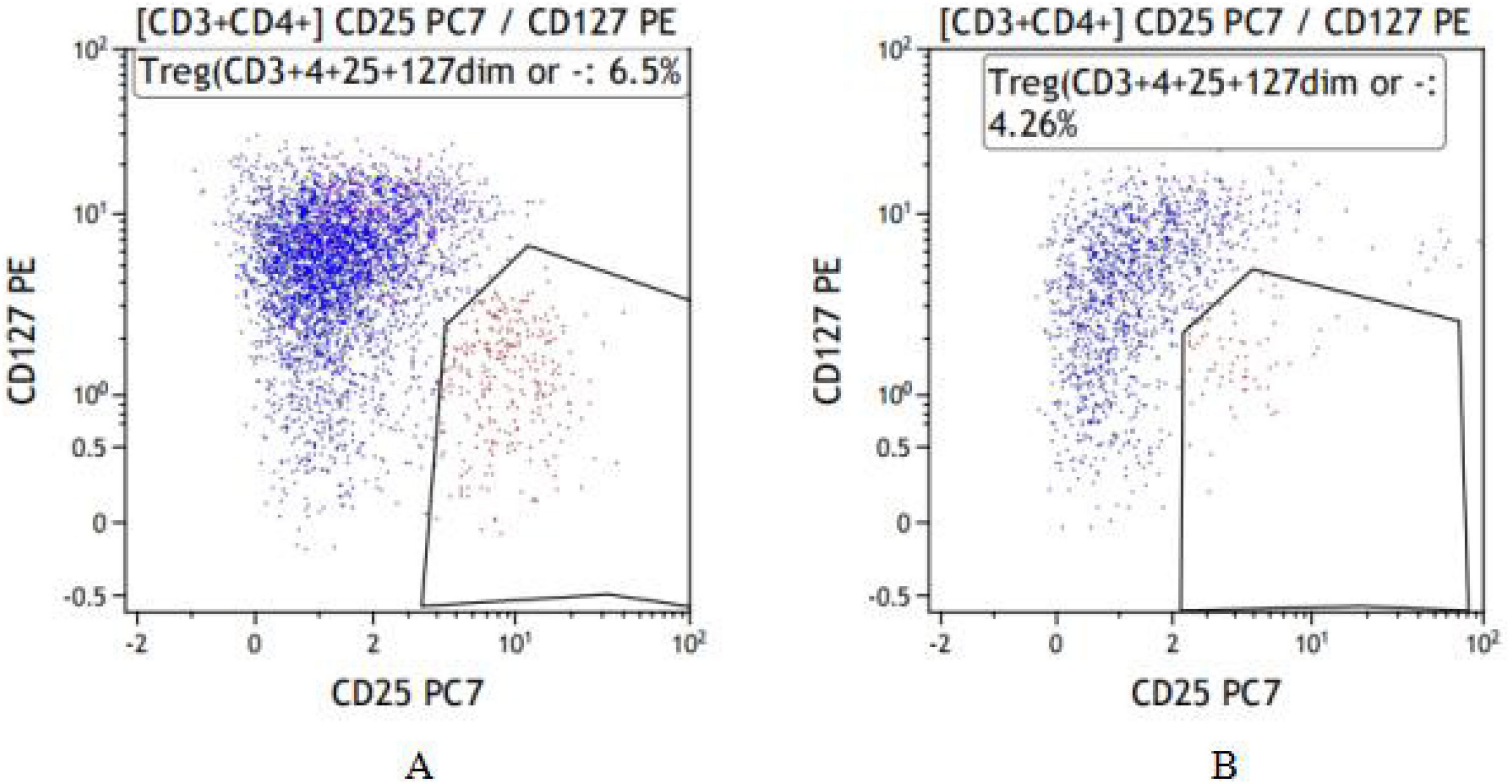
Typical flow cytometry plots of Treg cells from the two groups. **(A)** Frail group. **(B)** Non-frail group.

**FIGURE 2:**
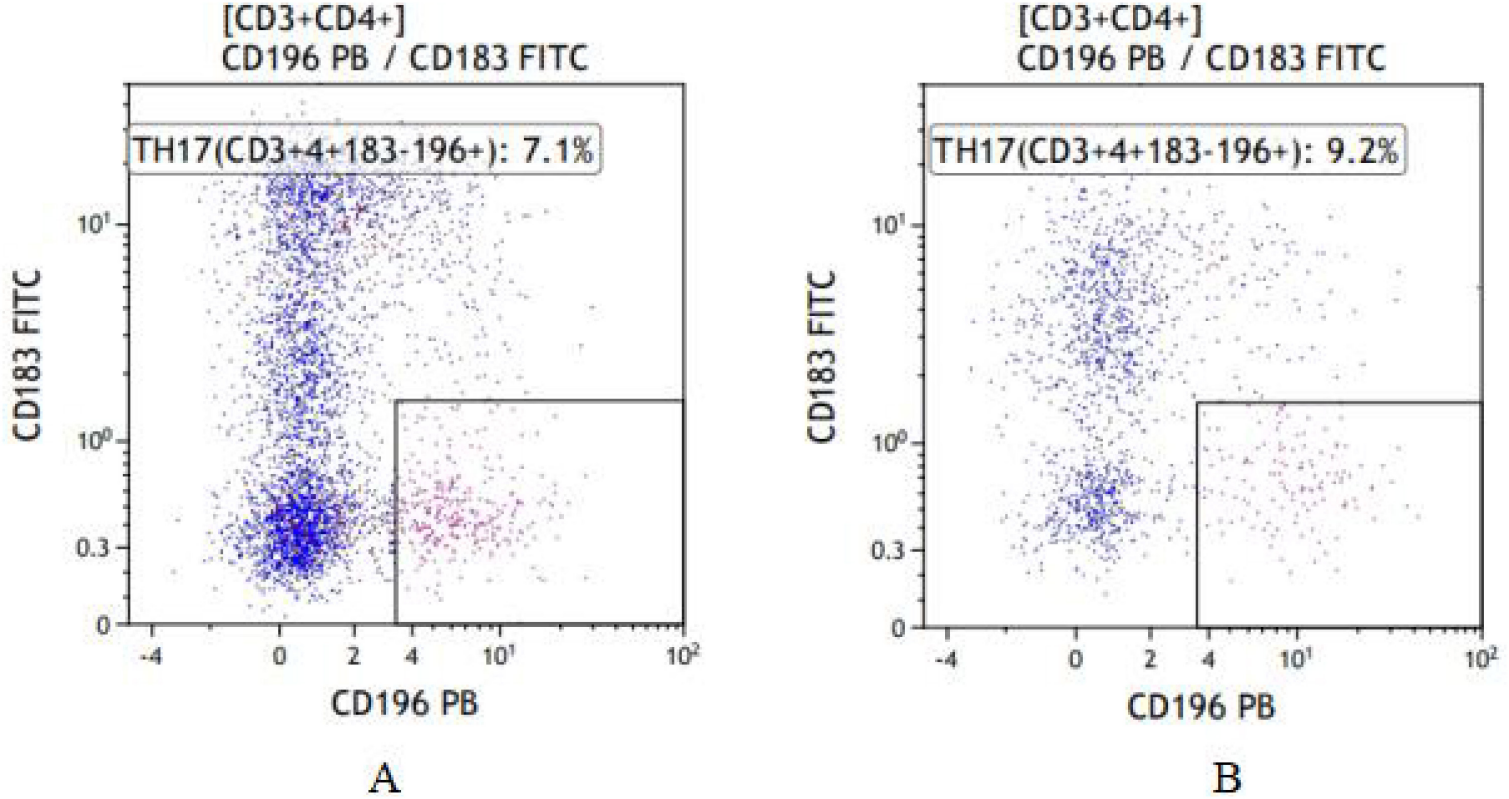
Typical flow cytometry plots of Th17 cells from the two groups. **(A)** Frail group. **(B)** Non-frail group.

The proportion of CD4^+^ T lymphocytes in the frail group was lower than that in the non-frail group (*P* < 0.05). The proportion of Treg cells among CD4^+^ T cells and the Treg/Th17 cell ratio were both higher in the frail group compared to the non-frail group (*P* < 0.05). There was no statistically significant difference in Th17 cell levels between the two groups (Figures 1, 2 and Table 4).

**TABLE 4:** Comparison of peripheral blood CD4^+^T, Th17, and Treg cell proportions and Th17/Treg ratio.

Groups	CD4^+^T (%)	Th17 (%)	Treg (%)	Treg/Th17
Frail group	0.33 (0.28,0.43)	7.10 (5.50,11.11)	6.50 (4.12,7.85)	0.90 (0.52,1.13)
Non-frail group	0.42 (0.36, 0.78)	8.75 (4.93, 11.91)	4.45 (3.31, 6.30)	0.56 (0.38, 0.73)
*Z*	–2.43	–7.09	–2.32	–2.84
*P*	<0.05	>0.05	<0.05	<0.05

### 3.5 Comparison of absolute counts of peripheral blood CD4^+^ T, Th17, and Treg cell

There were no statistically significant differences in the absolute counts of these cells between the two groups (*p* > 0.05), indicating that the observed increase in the proportion of Treg cell and the elevated Treg/Th17 ratio in the frail group primarily reflect compositional changes within the CD4^+^ T cell compartment, rather than differences in overall cell numbers. Thus, subsequent analyses focus on proportions and ratios to more accurately reveal the state of immune balance (Table 5).

**TABLE 5:** Comparison of absolute counts of peripheral blood CD4^+^ T, Th17, and Treg cell.

Groups	CD4^+^T (×10^9/L)	Th17 (×10^9/L)	Treg (×10^9/L)
Frail group	0.28 (0.16, 0.38)	0.019 (0.010, 0.031)	0.013 (0.009, 0.030)
Non-frail group	0.34 (0.23, 0.52)	0.032 (0.016, 0.047)	0.018 (0.009, 0.021)
*Z*	–1.607	–1.925	–0.333
*P*	>0.05	>0.05	>0.05

### 3.6 Comparison of TGF-β, IL-10, and IL-17A

The levels of TGF-β were significantly higher in the frail group compared to the non-frail group, while IL-17A levels were significantly lower (*P* < 0.05). No statistically significant difference was observed in IL-10 levels between the two groups (Table 6).

**TABLE 6:** Comparison of TGF-β, IL-10, and IL-17A.

Groups	TGF-β (pg/mL)	IL-10(pg/mL)	IL-17A(pg/mL)
Frail group	198.61 (124.12, 221.28)	31.45 (21.79, 36.27)	8.29 (7.05, 13.60)
Non-frail group	121.44 (97.70, 186.07)	25.26 (19.96, 31.97)	12.94 (9.15, 17.31)
*Z*	–2.58	–1.15	–2.46
*P*	*P*<0.05	*P*>0.05	*P*<0.05

### 3.7 Correlation between TGF-β, IL-10, IL-17A levels, and Th17/Treg cell proportions

**FIGURE 3:**
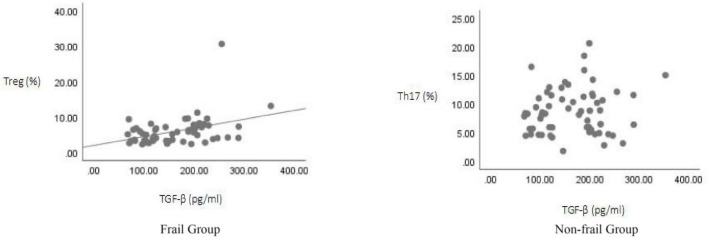
Correlation analysis of TGF-β levels with Th17 and Treg cell proportions.

**FIGURE 4:**
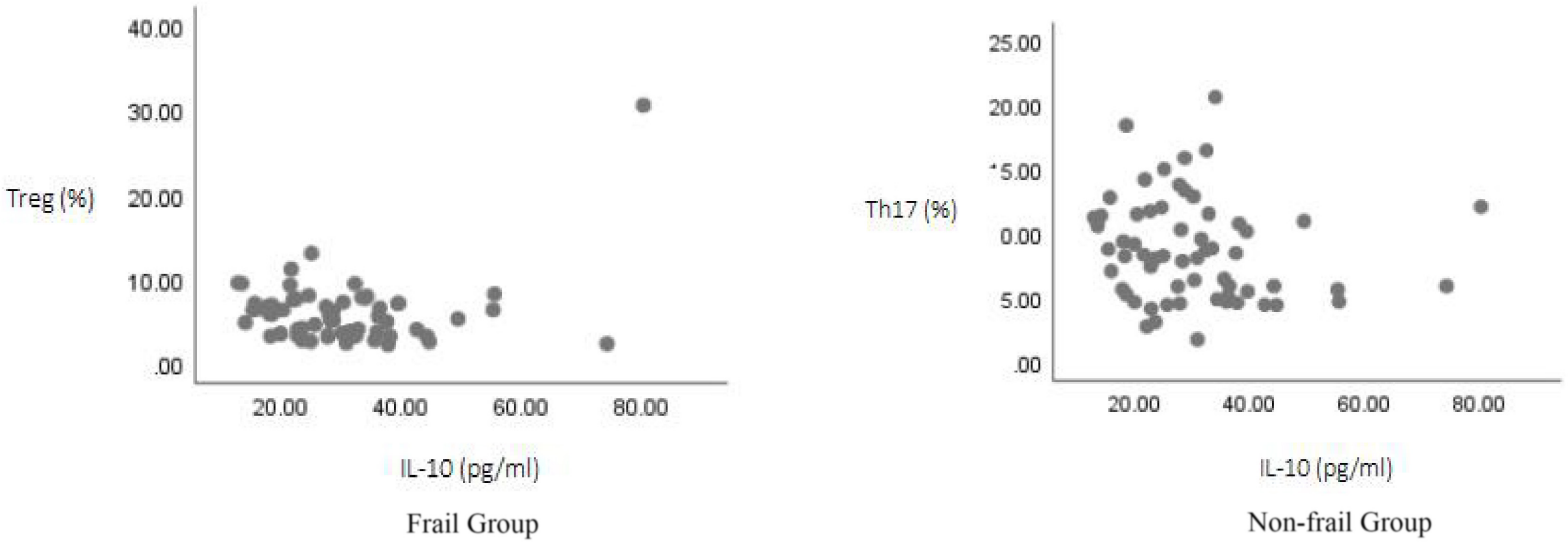
Correlation analysis of IL-10 levels with Th17 and Treg cell proportions.

**FIGURE 5:**
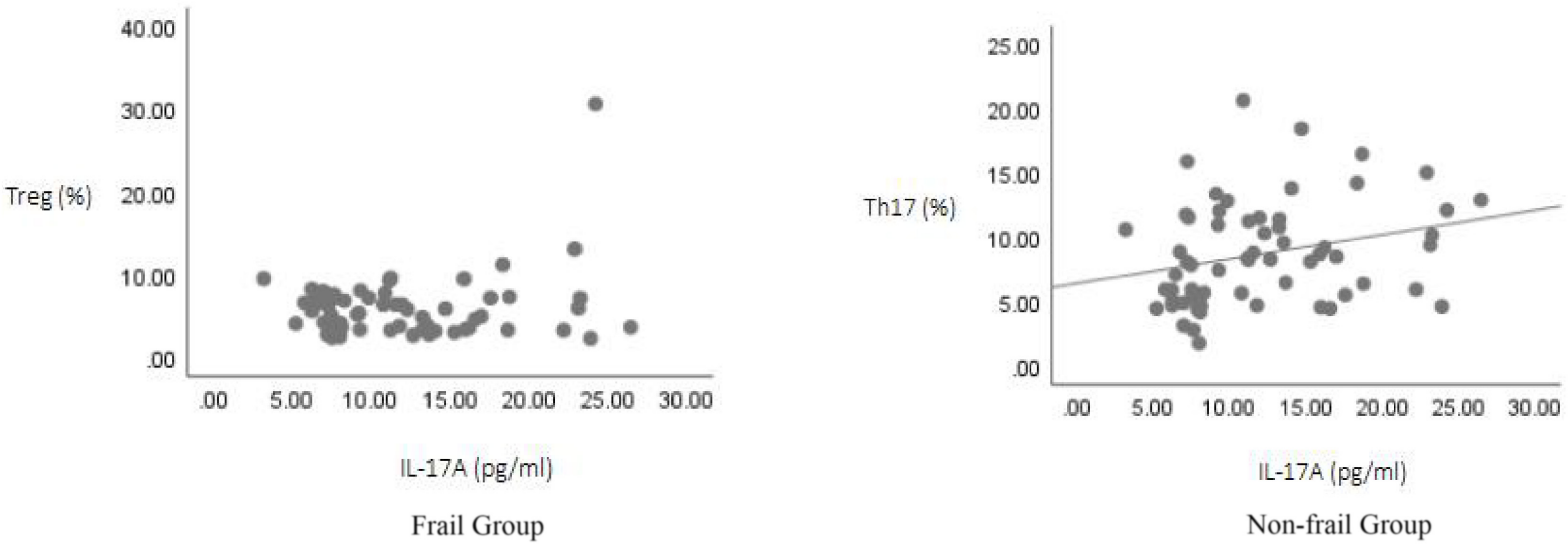
Correlation analysis of IL-17A levels with Th17 and Treg cell proportions.

Correlation analysis was performed between TGF-β, IL-10, and IL-17A and the proportions of Th17 and Treg cell for all study subjects using Spearman’s test. The results showed: TGF-β levels were positively correlated with Treg cell proportion (*r* = 0.416, *p* < 0.05), but showed no significant correlation with Th17 cell proportion (*r* = 0.059, *p* > 0.05); IL-17A levels were positively correlated with Th17 cell proportion (*r* = 0.316, *p* < 0.05), but showed no significant correlation with Treg cell proportion (*r* = –0.107, *p* > 0.05); IL-10 levels showed no significant correlation with either Treg cell proportion (*r* = –0.191, *p* > 0.05) or Th17 cell proportion (*r* = –0.199, *p* > 0.05) (Figures 3–5).

### 3.8 Multifactorial linear stepwise regression analysis of factors influencing the Treg/Th17 cell ratio

After adjusting for factors such as age, gender, CCI score, PSI score, and partial laboratory test results, frailty remained an independent factor influencing the Treg/Th17 cell ratio (β = 0.334, *P* < 0.05). A multivariate logistic regression equation was constructed by including age, gender, CCI score, PSI score, Treg/Th17 cell ratio, CD4^+^ T lymphocyte proportion, creatinine, albumin, and NT-proBNP. The results showed that advanced age, high CCI score, high Treg/Th17 cell ratio, low albumin, and female gender were independent risk factors for community-acquired pneumonia combined with frailty in the elderly (Tables 7, 8).

**TABLE 7:** Multiple linear stepwise regression analysis of factors influencing the Treg/Th17 cell ratio.

Variables	β	SE	t	*P*
Frailty	0.334	–0.123	2.700	0.009

**TABLE 8:** Multivariate logistic regression analysis of risk factors for frailty in elderly patients with CAP.

Variables	B	SE	Wald	Sig	OR(Exp B)	*P*
Gender	–4.442	1.812	6.006	0.014	0.012	<0.05
Age	0.349	0.142	6.058	0.014	1.418	<0.05
CCI	1.095	0.536	4.174	0.041	2.988	<0.05
PSI	–0.040	0.041	0.939	0.333	0.961	>0.05
Treg/Th17 ratio	4.301	1.770	5.907	0.015	73.784	<0.05
CD4^+^T lymphocyte	–8.577	5.626	2.324	0.127	0.000	>0.05
Creatinine	0.013	0.012	1.126	0.289	1.013	>0.05
Albumin	–0.362	0.165	4.814	0.029	0.696	<0.05
NT-proBNP	0.000	0.000	0.445	0.505	1.000	>0.05

## 4 Discussion

CAP is one of the most critical diseases affecting the quality of life and mortality rate among the elderly. In China, approximately 2.4 per 10,000 individuals aged 65–69 die from CAP, a figure that increases nearly 36-fold among those aged 85 and above (15). Clinically, elderly CAP patients with concomitant frailty are not uncommon. In this study, the prevalence of frailty among elderly CAP patients was 54.10%, significantly higher than the frailty prevalence in the general elderly community population (5.6–11.1%). Furthermore, consistent with previous studies, this study identified advanced age and female gender as independent risk factors for frailty in CAP patients (16). According to the PSI score, the severity of pneumonia was higher in the frail group compared to non-frail patients. Compared to the widely used PSI score, a patient’s frailty status better reflects their overall functional state prior to acute hospitalization, providing a robust basis for clinicians to assess disease severity, prognosis, and make clinical decisions (17). In a follow-up study of 521 elderly pneumonia patients with an average age of 80, Kanji Yamada et al. confirmed that frailty is an independent risk factor for increased 6-month mortality and readmission rates among these patients. Recurrent pneumonia was the most common cause of readmission, accounting for 45.7% of cases (18). In this study, all five patients who died within 30 days were from the frail group, with a mortality rate of 15.15%. Consistent with other research, the frail group also had significantly longer hospital stays and higher medical costs compared to non-frail patients, further straining healthcare resources.

In this study, creatinine and NT-proBNP levels were higher in the frail group compared to the non-frail group, indicating poorer functional status of the heart and kidneys in frail patients, which may suggest a higher burden of comorbidities. Surveys indicate that the prevalence of comorbidities among individuals aged 65 and older exceeds 55%, and among frail patients, this figure rises to as high as 72%. Additionally, the presence of comorbidities nearly doubles the risk of developing frailty (19, 20). Frailty and comorbidities are mutually reinforcing and are both associated with increased risks of disability, hospitalization, and mortality in the elderly. The Charlson Comorbidity Index (CCI) is currently regarded as the gold standard in clinical research for assessing patient comorbidities, as it accurately reflects individual disease burden. Previous studies have demonstrated that CCI is correlated with disease prognosis (21). In this study, CCI scores were significantly higher in frail patients compared to non-frail patients, and CCI was identified as an independent risk factor for frailty in elderly patients CAP. Therefore, comprehensive management of comorbidities in elderly patients with CAP should be prioritized. Furthermore, albumin levels in both groups of this study were below normal ranges, with frail patients exhibiting even lower levels than non-frail patients. Hypoalbuminemia was identified as an independent risk factor for frailty in elderly CAP patients. Other studies have also found that hypoalbuminemia is an independent risk factor for pneumonia in the elderly. Serum albumin levels can, to some extent, reflect an individual’s nutritional and metabolic status over a period of time. Thus, attention to the nutritional status of the elderly and early intervention for malnutrition may help reduce the risks of both pneumonia and frailty (22).

Th17 cells can trigger inflammatory responses by secreting pro-inflammatory cytokines such as IL-17A, IL-17F, IL-22, and TNF-α, thereby participating in host immune defense and pathogen clearance. Among these, IL-17A has been demonstrated to play a protective role in pneumonia caused by various extracellular or intracellular pathogens, such as Klebsiella pneumoniae and Mycoplasma pneumoniae. Its mechanisms include promoting neutrophil recruitment, stimulating antimicrobial peptide secretion, and enhancing the expression of cell adhesion molecules like ICAM-1 (23). However, some studies have also found that Th17 cells and IL-17A-mediated immune responses can exacerbate lung tissue damage (24). Treg cells are components in the regulation of immune responses. They exert immunosuppressive effects by secreting inhibitory cytokines such as IL-10, IL-35, and TGF-β, and are involved in maintaining self-tolerance, anti-tumor responses, transplantation immunity, and infection processes. Low expression of CD127 is a specific marker for Treg cells, and the CD4^+^CD25^+^CD^127–/low^ phenotype is commonly used to identify Treg cells (14). Treg cells play varying roles across different experimental conditions or disease processes. On one hand, they can exert a protective function by suppressing excessive immune responses and mitigating inflammation-induced tissue damage. On the other hand, the inhibition of immune function may facilitate pathogen dissemination, hinder infection containment, and lead to disease progression or exacerbation. Treg and Th17 cells interact and jointly participate in maintaining the body’s homeostasis and normal immune responses. Disruption of the Treg/Th17 balance can have adverse effects on the body and is involved in the development of various lung diseases, including pneumonia. During infection, the Treg/Th17 ratio undergoes dynamic changes, playing different roles at various stages of the disease. In animal models of pulmonary infection, the proportion of Th17 cells rapidly increases in the early stages of infection, leading to a decrease in the Treg/Th17 ratio. This triggers a strong pro-inflammatory response, generating protective immunity against the infection. As the infection progresses, the proportion of Treg cells gradually rises, and by the mid to late stages of infection, Treg cells become dominant, exerting immune homeostatic functions to prevent excessive tissue damage.

Previous studies have observed a significant decrease in the Treg/Th17 cell ratio in the early stages of infected patients, which triggers a strong pro-inflammatory response in the body and plays an anti-infective defensive role (25). In frail elderly patients with CAP, we observed a characteristic pattern of immune imbalance in peripheral blood, marked by an increased proportion of Treg cells and an elevated Treg/Th17 ratio, while the proportion of Th17 cells showed no significant change. It is important to note that this study focused on the Treg/Th17 ratio and the proportion of Tregs within CD4^+^ T cells, rather than absolute cell counts. In the context of immunosenescence and chronic inflammation, functional immune imbalance is often characterized by a dysregulation in the relative proportions of key cellular subsets, rather than changes in their absolute numbers. Proportional metrics better reflect the internal balance between regulatory and effector functions within the immune system and are less influenced by inter-individual variations in total lymphocyte counts. Our data support this view: Although there were no significant differences in the absolute counts of CD4^+^ T cells, Tregs, or Th17 cells between the frail and non-frail groups, frail patients exhibited a significant shift toward Treg predominance. This aligns with the theoretical model of immunosenescence. Therefore, proportions and ratios represent more sensitive and biologically meaningful indicators for assessing immune imbalance.

This immune phenotype suggests a deeper disturbance of immune homeostasis that is closely related to frailty itself rather than to infection alone. Frailty is not simply a consequence of aging but a clinical syndrome associated with chronic systemic inflammation and immune dysregulation. Recent proteomic studies have shown that frail individuals display a distinct pro-inflammatory protein profile, with persistent and poorly controlled activation of pathways such as IL-6/JAK/STAT (26). This state of inflammaging creates a dysregulated background for T-cell differentiation and function. Under such conditions, chronic inflammatory signals may act as a continuous stimulus that drives a compensatory expansion of immunosuppressive Tregs; however, in frailty this expansion may be inefficient and accompanied by functional exhaustion, resulting in an apparent increase in Treg proportion but impaired regulatory capacity. In contrast, effective Th17 responses require coordinated and robust signaling inputs, particularly cytokines such as IL-23 that are essential for Th17 expansion and maintenance (27). The disorganized and low-efficiency inflammatory milieu seen in frailty may fail to provide these key signals, thereby limiting Th17 activation. In addition, frailty-related metabolic dysregulation may further influence T-cell fate decisions at a more fundamental level. Recent studies have identified phosphoglycerate mutase 1 (PGAM1) as a metabolic checkpoint that supports Treg differentiation, whereas its inhibition shifts CD4^+^ T cells toward IL-17 production (28). It is therefore plausible that frailty, through altered immune metabolic networks, maintains an intrinsic metabolic state that favors Treg differentiation and biases lineage commitment. Taken together, this Treg-dominant and Th17-restricted immune landscape places frail patients in a dilemma when faced with acute infections such as CAP: functionally compromised Tregs are insufficient to control excessive inflammation, while inadequate Th17 responses impair pathogen clearance. The coexistence of dysregulated immune control and weakened antimicrobial defense may help explain the greater disease severity, higher complication rates, and poorer outcomes observed in frail CAP patients, linking frailty as a clinical syndrome to a specific immune cell phenotype and its adverse clinical consequences.

Although direct functional assays of Treg and Th17 cells were not performed in this study, the combined assessment of phenotypically defined T-cell subsets and their associated cytokines provides biologically meaningful insight into the functional orientation of the Treg/Th17 axis *in vivo*. In frail elderly patients with acute infection, extensive *ex vivo* functional assays are often constrained by patient vulnerability and limited sample availability. Therefore, relative cell proportions together with signature cytokines are widely used as surrogate indicators of immune regulatory balance in clinical studies.

In this context, the cytokine profiles observed in our study offer important mechanistic clues underlying the altered Treg/Th17 balance in frail patients with CAP. We observed significantly elevated TGF-β levels in the frailty group, which may contribute to the increased proportion of Treg cells and the elevated Treg/Th17 ratio. However, it should be noted that TGF-β is derived from multiple cellular sources, including not only Treg cells but also activated macrophages, neutrophils, platelets, as well as epithelial cells and fibroblasts involved in tissue injury and repair. Accordingdly, the increased TGF-β levels observed in frail patients are more likely to reflect the combined effects of frailty-associated chronic inflammation and acute infection rather than being solely attributable to Treg expansion. In parallel, IL-17A levels were reduced in the frailty group and showed a positive correlation with Th17 cell proportions, consistent with an attenuated Th17-mediated inflammatory defense. During the early phase of infection, adequate IL-17A production is critical for neutrophil recruitment and the initiation of mucosal immune defenses. Lower IL-17A levels in frail patients may reflect an impaired Th17 response, which may partially explain their suboptimal infection control and poorer clinical outcomes. Notably, IL-10 levels did not differ between groups and were not significantly associated with Treg proportions, suggesting that the immunoregulatory effects of circulating Treg cells may not be predominantly mediated through IL-10. Treg cells are known to exert suppressive functions through multiple mechanisms, including cell contact–dependent pathways such as CTLA-4 and the secretion of other inhibitory mediators, including TGF-β, IL-35, and adenosine. Moreover, the dissociation between IL-10 levels and Treg proportions highlights that Treg functional status cannot be fully characterized on the basis of cell frequencies and a limited panel of cytokines alone. Taken together, the cytokine findings indicate a shift toward Treg predominance in frail elderly patients with CAP, accompanied by reduced Th17-associated inflammatory activity. These alterations are best interpreted as reflecting changes in immune balance rather than preserved or enhanced cellular function. Future studies incorporating direct functional assays will be important to further clarify the contribution of Treg and Th17 cells to immune dysregulation associated with frailty.

In any case, this study provides novel evidence indicating an imbalance in Treg/Th17 cells in frail elderly patients with CAP, a finding that may be associated with their poorer prognosis and highlights the potential relevance of immune-targeted interventions.

## 5 Conclusion

The prevalence of frailty is notably high among elderly patients with CAP. Frail patients tend to have more comorbidities, present with more severe conditions, exhibit poorer prognoses, require longer hospital stays, and incur higher medical costs. Timely identification of frailty in elderly patients with CAP is of significant importance for disease assessment, treatment decision-making, and improving clinical outcomes.

Frail elderly patients with CAP exhibit an imbalance in Treg/Th17 cells compared to non-frail patients. This specific dysregulation of immune cell subsets may be closely associated with the poor prognosis observed in frail elderly CAP patients. Monitoring alterations in immunosenescence-related T-cell subsets in these patients is critical for prognosis evaluation and may provide novel insights and approaches for immunotargeted therapy in elderly patients with CAP.

Cytokines play a vital role in the differentiation, development, and functional expression of Treg and Th17 cells. In elderly patients with CAP, the proportions of Treg and Th17 cells largely align with the trends in their respective related cytokine levels, namely TGF-β and IL-17A. This suggests that dysregulated cytokine expression may contribute to the development of Treg/Th17 imbalance in frail elderly patients with CAP, offering new evidence to further elucidate the regulatory mechanisms underlying Treg/Th17 balance.

## 6 Limitation

Several limitations should be acknowledged when interpreting these findings. First, the cross-sectional design limits causal inference and precludes assessment of dynamic changes in immune parameters during the course of CAP. Although an association between frailty and Treg/Th17 imbalance was observed, longitudinal studies are required to clarify whether this immune profile precedes or results from frailty progression and disease severity. Second, the modest sample size from a single center may limit generalizability and reduce the statistical power for detecting weaker associations. While the primary immunological comparisons showed robust effect sizes, the results of multivariate analyses should be interpreted cautiously and warrant validation in larger, multicenter cohorts. Third, frailty was assessed using the FRAIL scale, a validated and widely used instrument that nonetheless relies partly on self-report and clinical judgment, which may introduce measurement variability. Future studies incorporating more comprehensive or objective frailty assessments could improve classification accuracy. Finally, this study focused on phenotypic proportions of Treg and Th17 cells without direct assessment of their functional activity. While the integration of cytokine profiles provides indirect evidence of functional dysregulation, cell frequencies alone cannot fully capture suppressive capacity, cytokine responsiveness, or metabolic fitness. Therefore, the observed increase in the Treg/Th17 ratio should be interpreted as a shift in immune balance rather than definitive proof of enhanced regulatory function. Future studies incorporating functional suppression assays, ex vivo stimulation experiments, transcription factor analysis, and metabolic profiling will be essential to clarify the precise role of Treg and Th17 cells in the pathophysiology of frailty-associated CAP.

## Data Availability

The original contributions presented in the study are included in the article/supplementary material, further inquiries can be directed to the corresponding author.
